# Pediatric Uveitis

**Published:** 2011-10

**Authors:** Ilknur Tugal-Tutkun

**Affiliations:** Department of Ophthalmology, Faculty of Medicine, Istanbul University, Istanbul, Turkey

**Keywords:** Pediatric Uveitis, Juvenile Idiopathic Arthritis, Pars Planitis, Behçet Uveitis, Tubulointerstitial Nephritis and Uveitis Syndrome

## Abstract

Uveitis is less common in children than in adults, and its diagnosis and management can be particularly challenging. Young children are often asymptomatic either because of inability to express complaints or because of the truly asymptomatic nature of their disease. Even in advanced cases, parents may not be aware of severe visual impairment until the development of externally visible changes such as band keratopathy, strabismus, or leukocoria. Therefore, the diagnosis is often delayed and severe complications may be seen at the time of initial visit. Young children may not be cooperative for a complete ocular examination and subtle findings of intraocular inflammation such as trace cells may be easily missed in the early stages of the disease. Children, in general, tend to have more severe and chronic intraocular inflammation that frequently results in ocular complications and visual loss. In children who present with amblyopia or strabismus, a careful examination is required to rule out uveitis as an underlying cause. Delayed and variable presentations cause a distinct challenge in the diagnosis of uveitis in children, furthermore differential diagnosis also requires awareness of etiologies which are different from adults. There are unique forms of uveitis and masquerade syndromes in this age group, while some entities commonly encountered in adults are rare in children.

## EPIDEMIOLOGY

In a population-based study in Finland, the yearly incidence of uveitis was found to be 4.3 per 100,000 in children and 27.2 per 100,000 in adults; prevalence rates were 27.9 and 93.1 per 100,000 respectively.^1^ In a study conducted in district hospitals in the UK, the incidence of pediatric uveitis increased with age from 3.15 per 100,000 children aged 0 to 5 years, to 6.06 per 100,000 of those between 11 and 15 years of age.^2^ Pediatric cases account for less than 10% of all cases of uveitis in large series reported from referral centers.

As in adults, there are variations in the geographic and ethnic distribution of various infectious and noninfectious uveitic entities in children. Table 1 shows the types of involvement and the most common diagnoses in pediatric uveitis series reported from various countries.^1^–^18^ Anterior uveitis is the most common form of involvement in almost all series. Up to 60% of patients have idiopathic uveitis. Juvenile idiopathic arthritis (JIA) associated anterior uveitis is the leading cause in series reported from Northern European countries and from the USA, whereas it is less frequent in Mediterranean and Middle Eastern countries as well as in India. In contrast, while Behçet’s disease accounts for 11% of pediatric uveitis in Turkey and 5–6% in Israel and Tunisia, it is very rare in European countries, the USA and India. Vogt-Koyanagi-Harada disease is distinctly more common in Saudi Arabia. Pars planitis has been reported as a distinct entity in some series, but was included among unspecified idiopathic cases in others. Therefore, it is difficult to compare the frequency of idiopathic pars planitis among published series. Among infectious causes of uveitis in children, toxoplasmosis is the most common etiology. Herpetic anterior uveitis is uncommon in childhood irrespective of geographic region. Tuberculosis has been reported in 3.9% of pediatric uveitis cases in India, where the disease is endemic.^14^

## DIAGNOSTIC ENTITIES

### JIA-associated Anterior Uveitis

JIA is the most common systemic association of pediatric uveitis. It is defined as arthritis of at least 6 weeks’ duration without any other identifiable cause in children younger than 16 years of age. The International League of Associations for Rheumatology (ILAR) has classified JIA into seven subtypes. Patients with systemic-onset JIA have extraocular manifestations such as fever, rash, hepatosplenomegaly, lymphadenopathy, and serositis. Uveitis is extremely rare in this subtype. The oligoarticular subtype is diagnosed when fewer than 5 joints are involved during the first 6 months of the disease. Chronic anterior uveitis is most commonly associated with oligoarticular JIA. This subtype is defined as extended oligoarthritis when more joints become involved after 6 months. Polyarticular JIA is diagnosed when 5 or more joints are affected during the first 6 months of the disease. Polyarticular JIA may be rheumatoid factor (RF) positive or negative. Uveitis is rare in the RF-positive group, however approximately 10% of patients with RF-negative polyarticular JIA develop uveitis. Psoriatic arthritis is a less common subtype of JIA which may be associated with chronic anterior uveitis in 10–20% of cases. Enthesitis-related arthritis typically occurs in older boys who are often HLA-B27 positive. Acute unilateral anterior uveitis is the typical form of ocular involvement in these patients. Patients with arthritis who do not fulfill any of these categories are classified as the “other” JIA subgroup.^19^, ^20^

Risk factors for ocular involvement in patients with JIA include female sex, oligoarticular arthritis, young age at onset of arthritis, antinuclear antibody (ANA) seropositivity and RF seronegativity. 21

Children with chronic anterior uveitis associated with JIA are typically asymptomatic and therefore routine ophthalmologic screening is essential for early diagnosis and timely treatment. Otherwise, affected subjects may present after development of serious ocular complications and severe visual impairment. Ocular condition at the first visit is the most significant predictor of visual outcome. Onset of uveitis before or early after the onset of arthritis has also been reported as a predictor of poor visual outcome. Screening guidelines have been developed^22^; patients with oligo- or polyarticular JIA with onset of arthritis at 6 years of age or younger, duration of arthritis 4 years or less, and ANA-seropositivity should be screened at 3-month intervals.

**Figure 1:**
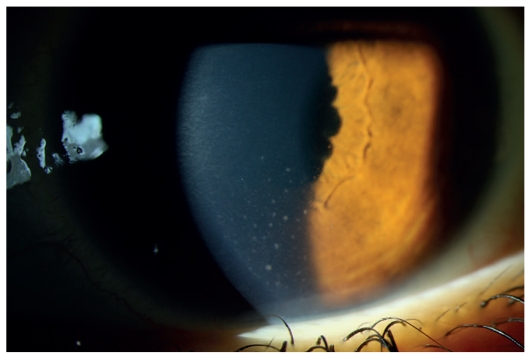
Slit lamp photograph shows medium-sized granulomatous keratic precipitates located inferiorly in a child with JIA-associated anterior uveitis.

**Figure 2:**
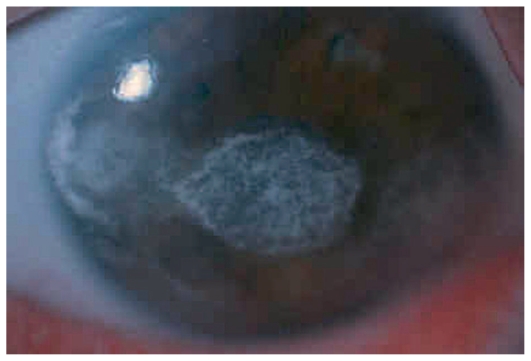
Diffuse illumination at the slit lamp shows severe band keratopathy in the left eye of a boy with JIA-associated anterior uveitis.

**Figure 3:**
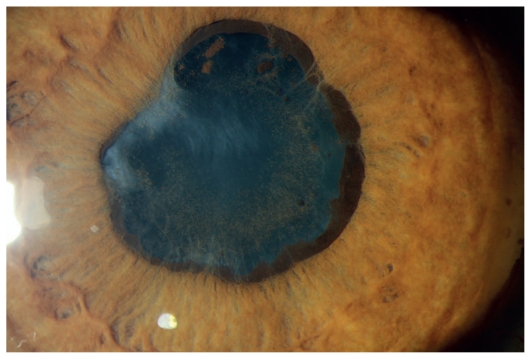
Slit lamp photography using diffuse illumination at high magnification shows seclusion of the pupil in a child with JIA-associated anterior uveitis.

JIA-associated anterior uveitis is typically bilateral, nongranulomatous, and has a chronic relapsing course. However, a granulomatous disease does not exclude the diagnosis (Fig. 1). Mutton-fat keratic precipitates (KPs) or iris nodules, reminiscent of sarcoidosis, have been reported in 24% of white and 56% of non-white patients.^23^ The most common complications of JIA-associated anterior uveitis include band keratopathy (Fig. 2), cataract, posterior synechiae (Fig. 3), glaucoma, maculopathy, hypotony and amblyopia.^24^,^25^ In a recent study, 84% of eyes with JIA uveitis were found to have maculopathy on optical coherence tomography.^26^ The presence of complications at the time of initial visit and high anterior chamber flare values by laser flare photometry are risk factors for development of new complications and poor visual prognosis.^25^,^27^,^28^ Early development of cataract requiring surgery has been associated with the presence of posterior synechiae at the time of diagnosis.^29^ Early immunomodulatory treatment reduces the risk of ocular complications and visual loss.^24^,^25^,^29^ Since high anterior chamber flare is associated with an increased risk of visual loss independent of anterior chamber cells,^28^ children with high flare should be treated aggressively with the aim of reducing flare to the minimum possible level.

Chronic anterior uveitis can also occur in children without systemic disease associations. In a cohort of 115 patients with chronic anterior uveitis, no significant association was found between the presence of systemic disease (JIA) and ocular complications or visual loss. On the other hand, elevated flare (≥20photons/msec) has been found to be a strong predictor of complications and visual loss.^30^ Laser flare photometry is therefore an essential tool in the follow-up of children with chronic anterior uveitis.

### Idiopathic Intermediate Uveitis (Pars Planitis)

According to the anatomic classification of uveitis by the Standardization of Uveitis Nomenclature (SUN) Working Group, the term “intermediate uveitis” defines a subset of uveitis where the vitreous is the primary site of inflammation. Pars planitis is a diagnostic term that defines a subset of idiopathic intermediate uveitis where there is snowbank or snowball formation.^31^ This disease typically affects children and adolescents. The association of intermediate uveitis with a systemic disease is very rare in children. Associations between idiopathic intermediate uveitis and HLA-DR2 and HLA-DR15 have been reported suggesting an immunogenetic predisposition.^32^,^33^ A strong association of multiple sclerosis with the same HLA antigens suggests a common genetic background.

Young children with pars planitis are usually asymptomatic and diagnosed during a routine ophthalmologic examination. Some children are diagnosed only after significant visual impairment or the development of complications that cause leukocoria or strabismus. Most children have bilateral symmetrical involvement.

**Figure 4:**
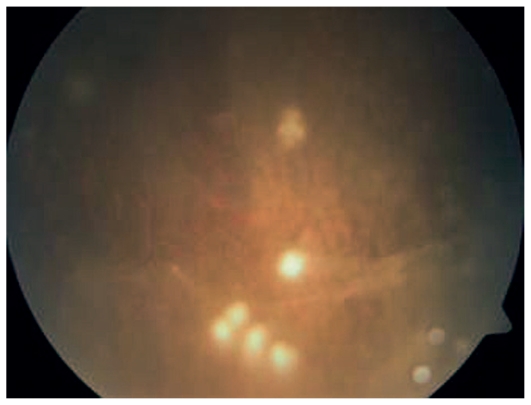
Fundus photograph shows inferiorly located snow ball opacities and vitreous veils in a child with pars planitis.

**Figure 5:**
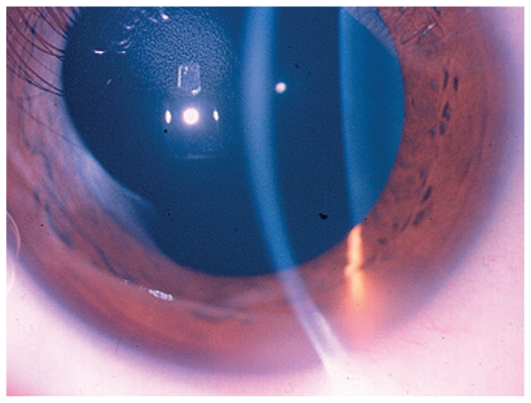
Slit lamp photograph shows peripheral corneal edema due to peripheral corneal endotheliopathy localized to the inferior-temporal cornea of the left eye in a child with pars planitis.

Typical clinical findings include mild to moderate anterior segment inflammation, diffuse vitreous cells and haze, and snowballs and snowbanks located inferiorly (Fig. 4). Band keratopathy, peripheral corneal endotheliopathy (Fig. 5), and posterior synechiae may be seen in childhood pars planitis but are very rare in adults. Optic disc edema and cystoid macular edema are the most frequent complications.^34^

**Figure 6:**
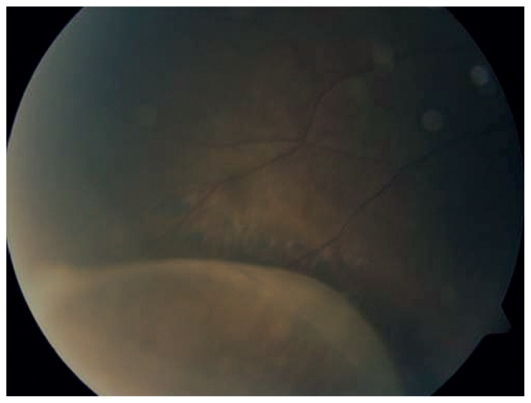
Fundus photograph shows inferior retinoschisis in a child with pars planitis.

**Figure 7:**
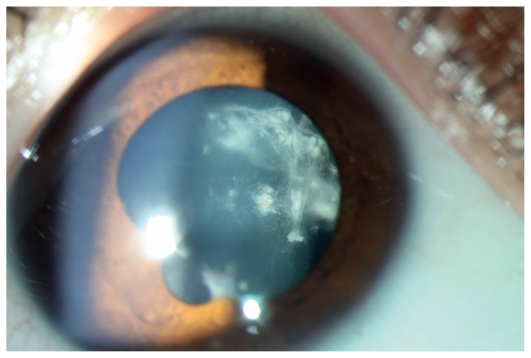
Slit lamp photograph shows dense vitreous condensation and opacities adherent to the posterior lens capsule in a child with pars planitis. Also note irregular pupil dilation due to posterior synechiae in this eye.

Neovascularization of the optic disc or associated with snowbanks may cause vitreous hemorrhage and is more common in children than adults with pars planitis. Inferior peripheral retinoschisis is another complication that develops almost exclusively in children (Fig. 6).^35^ Dense vitreous condensation may cause leukocoria, sometimes mistaken for cataracts (Fig. 7). However, posterior subcapsular cataract may also develop early in the course of the disease.^36^ Although vitreous haze and cataracts may cause amblyopia in a young child with pars planitis, cystoid macular edema is the leading cause of visual morbidity.^34^,^35^ In a long-term follow-up study, visual prognosis was reported to be good despite the high rate of ocular complications in children with pars planitis. However, a recent study has shown that children with onset of disease at 7 years of age or younger were at higher risk of complications such as cataract, glaucoma, and vitreous hemorrhage, and had worse visual prognosis than older children.^37^ Periocular corticosteroid injections and short-term oral steroids are used in patients who have sight-threatening intraocular inflammation, especially cystoid macular edema. Repeated corticosteroid injections may induce ocular hypertension.^34^ Methotrexate and cyclosporine may be used as steroid-sparing agents. Children with severe intermediate uveitis of the pars planitis type may even require treatment with biologic agents. In a retrospective analysis of 20 children with refractory uveitis treated with infliximab, 8 had idiopathic pars planitis.^38^ Infliximab infusions could be discontinued in 2 of them after 7 and 12 infusions because remission could be sustained with only antimetabolites. Intraocular inflammation was controlled in all of the remaining 6 pars planitis patients with repeated infliximab infusions and a concomitant antimetabolite agent without any need for oral corticosteroids.^38^

### Behçet Uveitis

The peak age of onset for Behçet disease is in the third or fourth decade of life. Although the onset of recurrent oral ulcers in childhood is not uncommon, patients typically fulfill the diagnostic criteria after the age of 16 years. There are no internationally accepted diagnostic criteria for childhood-onset Behçet disease. In a recent international registry of patients suspected of pediatric Behçet disease, the presenting symptom was isolated recurrent oral ulcers in 83%, and the diagnosis was confirmed by an expert committee in 62% of registered cases. Uveitis was less common (34%) in this group than in adults.^39^ From an ophthalmological point of view, pediatric Behçet uveitis is defined as onset of uveitis at 16 years of age or younger irrespective of the age for fulfilling the diagnostic criteria. There may be variations in the prevalence of pediatric Behçet disease in different ethnic groups or geographic regions. In an international retrospective survey, more than half of the children with Behçet uveitis were from Middle Eastern countries as compared to only 2.4% from East/South Asia, in contrast 19.2% of adults belonged to the latter ethnic group.^40^ However, several sources of bias may have confounded the results of this survey. Even in Turkey, one of the countries with the highest prevalence of Behçet disease, only 3.5% of patients with Behçet uveitis are in the pediatric age group [unpublished data from the Turkish Registry of Uveitis (BUST study)].

Mean age at onset of pediatric Behçet uveitis is in late childhood (10–15 years).^41^–^44^ There is a male predominance in the pediatric age group, similar to adult-onset Behçet uveitis.^39^–^44^ A positive family history has been reported in 20–47% of pediatric cases from endemic areas, implying the role of genetic factors in the early onset of the disease.^39^,^41^,^42^,^45^ Otherwise, the clinical picture in children is not different from adults. The majority of patients have bilateral involvement and recurrent panuveitis with retinal vasculitis.^40^,^41^,^43^,^44^ Cataract, intraocular pressure elevation, macular edema or maculopathy, and optic atrophy are the most common complications.^41^,^43^–^45^ While visual prognosis has been reported to be better than adults in some series^40^,^43^, individual variability of the disease course, response to treatment and visual outcome has been reported by others.^41^,^44^,^45^ Children with posterior segment involvement require immunosuppressive therapy. Biologic agents are used in patients refractory to conventional immunosuppressive therapy. In a series of seven patients with corticosteroid dependent Behçet uveitis, treatment with interferon-alpha showed a corticosteroid-sparing effect with induction of remission in five patients; remission was sustained in four of them even after discontinuation of treatment in three.^46^

### Tubulointerstitial Nephritis and Uveitis (TINU) Syndrome

TINU is an uncommon syndrome accounting for 1.7% of all cases of adult uveitis.^47^ However, it is a more common cause of uveitis in children and adolescents with a median age of onset of 15 years.^48^ Diagnosis is based on the concurrence of acute tubulointerstitial nephritis (TIN) and bilateral acute anterior uveitis.^49^ Uveitis may not occur simultaneously, but may precede or more commonly follow renal disease.^48^ For a definite diagnosis, a renal biopsy may be performed. A clinical diagnosis of TIN is often based on the presence of systemic symptoms, including fever, weight loss, abdominal and flank pain, and arthralgia associated with renal dysfunction evidenced by increasing urea and creatinine levels, proteinuria, microhematuria, and glycosuria.^49^ An elevated urinary beta-2-microglobulin level has been reported as a very helpful laboratory marker.^48^–^50^

The typical presentation of TINU is an acute bilateral anterior uveitis, often with pain and photophobia. While topical corticosteroids may be sufficient in such cases, systemic corticosteroids and even immunosuppressive therapy may be required for renal disease or for patients who develop chronic recurrent anterior uveitis leading to ocular complications. Atypical cases with posterior or panuveitis may also require more aggressive treatment. Visual prognosis is generally good.^48^–^50^ In a recent retrospective study of 26 Finnish children with TIN, bilateral anterior uveitis was found in 12 subjects (46%).^51^ No correlation was found between renal disease and uveitis which preceded TIN by one month in one patient and followed TIN by 2 weeks to 15 months in others. Four of the 12 patients (33%) developed chronic uveitis and remission was achieved only after anti-tumor necrosis factor (TNF) treatment in one of them.^51^

### Sarcoidosis

Childhood sarcoidosis is a rare multisystemic granulomatous inflammatory disorder. While older children may present with pulmonary involvement, young children typically present with a triad of arthritis, skin lesions and uveitis. Serum angiotensin-converting enzyme levels may be misleading because children tend to have higher levels than adults.^52^ Although a biopsy specimen showing non-caseating granulomatous inflammation is required for a definite diagnosis, the clinical diagnosis of ocular sarcoidosis can be made, at least in older children, based on typical signs of ocular disease and presence of laboratory abnormalities.^53^

Anterior uveitis is the most common type of involvement in children with sarcoidosis. Granulomatous KPs, iris nodules, and peripheral and broad-based posterior synechiae are typical findings. Chronic uncontrolled anterior uveitis may lead to complications such as band keratopathy cataracts, and glaucoma. Sometimes it is difficult to differentiate this clinical picture from JIA-associated uveitis. Inflammation of the posterior segment in the form of retinal vasculitis or multifocal choroiditis can be seen in sarcoidosis; however, intraocular inflammation is typically confined to the anterior segment in JIA-associated uveitis although posterior segment findings may develop as a complication of uncontrolled anterior uveitis.

Familial juvenile systemic granulomatosis, also known as Blau syndrome, has an autosomal dominant mode of inheritance and is characterized by granulomatous polyarthritis, skin rash and acute granulomatous anterior uveitis resembling sarcoidosis in young children.^54^,^55^ Since Blau syndrome is clinically indistinguishable from early-onset sarcoidosis, family history is an essential component in the assessment of children who present with these manifestations. The genetic mutation for both has been shown to involve the CARD15/NOD2 gene on chromosome 16q12, inherited in an autosomal dominant pattern in Blau syndrome but arising de novo in infantile sarcoidosis.^56^

## TREATMENT OF NONINFECTIOUS UVEITIS IN CHILDREN

Exclusion of an infectious cause of uveitis and masquerade syndromes is of utmost importance before the administration of nonspecific anti-inflammatory and immunomodulatory treatment. Corticosteroids remain first-line treatment for noninfectious uveitis in children. Topical corticosteroids are initially used for treatment of anterior segment inflammation. Periocular or subtenon corticosteroid injections may be used for treatment of intermediate or posterior uveitis, especially in unilateral cases or for the treatment cystoid macular edema. Prolonged use of topical corticosteroids and repeated periocular injections are associated with a higher risk of ocular complications in children.^57^–^59^ Intraocular pressure elevation and steroid-induced glaucoma occur more rapidly and may be refractory to treatment in children. It is difficult to detect and monitor intraocular pressure elevation especially in young, noncompliant children. Multiple periocular injections may also result in systemic corticosteroid complications.^60^ Intravitreal injections may prevent systemic side effects, but potential risks and rate of ocular complications are higher.

Systemic corticosteroids are used only for short-term treatment in children because of significant systemic side effects associated with their prolonged use, including cushingoid status, growth retardation, weight gain, hypertension, osteoporosis, gastrointestinal disturbance, psychosis and electrolyte imbalance. The usual induction dose of oral prednisolone is 1–2mg/kg. Intravenous pulse methyl-prednisolone 30mg/kg may be preferred when more rapid and potent action is needed. Patients who do not adequately respond to high dose corticosteroids will need immunosuppressive treatment. For patients who become corticosteroid-dependent, a corticosteroid-sparing immunosuppressive agent is administered. In patients who present with serious ocular complications and risk factors for development of new complications, immunosuppressive agent(s) combined with corticosteroids may be started at initial visit.

Methotrexate is the most widely used first-line immunomodulatory agent in children with uveitis because of its long-term safety profile in this age group. In a recently reported series of JIA-associated uveitis, improvement of inflammation was achieved in 82% of patients treated with methotrexate for at least 3 months.^61^ A longer duration of inactivity during methotrexate therapy was found to be associated with a lower risk of relapse after discontinuation of this agent.^61^ Second-line immunosuppressive agents include azathioprine, cyclosporine and mycophenolate. Alkylating agents, such as cyclophosphamide and chlorambucil, are generally avoided in children because of serious potential side effects. These agents have been used in children with emergency situations such as systemic lupus erythematosus or other life-threatening vasculitides.

Anti-TNF agents are used in patients who fail to respond to conventional immunosuppressive therapy and are at a high risk of visual loss. Both infliximab and adalimumab have been successfully used for treatment of resistant pediatric uveitis.^38^,^62^–^69^ The convenience of subcutaneous administration, stable serum concentrations, and more favorable safety profile are advantages of adalimumab over intravenous infliximab infusions for treatment of uveitis in children. However, the fast-onset and potent anti-inflammatory effect of infliximab may be desirable for immediate control of intraocular inflammation, for example in children who need emergency ocular surgery.^38^ Another anti-TNF agent, etanercept, which is effective in the treatment of rheumatic disease in children, is not recommended for treatment of uveitis. Smith et al reported no significant difference between placebo and etanercept in a randomized controlled trial.^70^ Furthermore, several cases of new-onset uveitis have been reported in patients receiving etanercept for rheumatologic disorders.^71^,^72^ In patients refractory to anti-TNF agents, alternative biologic agents may be tried such as anakinra, daclizumab, abatacept, rituximab, and tocilizumab.^57^,^73^–^75^ Experience with these agents is currently limited to small series of selected patients. Furthermore, potential serious adverse effects such as an increased risk of opportunistic infections and malignancies should be carefully weighed against benefits of biologic therapy in this particular age group who have long life expectancy.

## Figures and Tables

**Table 1:** Pediatric uveitis series reported from various countries

Authors/ country	Year/N	Anterior uveitis	Intermediate uveitis	Posterior uveitis	Panuveitis	Idiopathic	JIA	Toxoplasmosis	Behçet’s disease	VKH	Herpetic uveitis
Tugal-Tutkun et al/USA [3]	1996/130	58.4%	20%	13.8%	7.6%	36.8%	41.5%	7.7%	0.7%	1.5%	2.2%
Pivetti-Pezzi/ Italy [4]	1996/267	33.3%	25.1%	26.6%	15%	54.3%	9.4%	11.6%	2.2%	1.5%	5.6%
Soylu et al/ Turkey [5]	1997/90	33.3%	8.9%	23.3%	34.4%	34.4%	3.3%	25.6%	11.1%	-	5.1%
Paivönsalo-Hietanen et al/ Finland [1]	2000/55	90.9%	1.8%	5.5%	1.8%	47.3%	36.3%	5.5%	-	-	5.5%
Stoffel et al/ Switzerland [6]	2000/70	57%	-	14%	29%	54%	34.3%	NA	1.4%	-	NA
De Boer et al/ Netherlands [7]	2003/123	36%	24%	19%	21%	53.7%	20%	10%	-	-	3.3%
Kadayifcilar et al/Turkey [8]	2003/219	43.4%	11.9%	31%	13.7%	36%	13.2%	21%	10.9%	0.5%	0.9%
Edelsten et al/ UK [2]	2003/249	70%	-	30%	-	44%	47%	2%	-	-	-
Azar et al/ Australia [9]	2004/40	66%	5.7%	13.2%	15.1%	60%	17.5%	2.5%	-	-	12.5%
Rosenberg et al/ USA [10]	2004/148	30.4%	27.7%	23.7%	18.2%	26.4%	23%	7.4%	0.7%	0.7%	2%
Kump et al/ USA [11]	2004/269	56.9%	20.8%	6.3%	16%	51.7%	33%	3.4%	0.3%	0.7%	1.5%
Ben Ezra et al/ Israel [12]	2005/276	13.4%	41.7%	14.1%	30.8%	25.4%	14.9%	7.2%	4.7%	1.1%	3.6%
Khairallah et al/ Tunisia [13]	2006/64	31.3%	31.3%	20.3%	17.2%	49.9%	6.2%	14.1%	6.2%	1.5%	6.2%
Rathinam et al/ India [14]	2007/616	59.9%	8.4%	11%	20.6%	32.5%	1.8%	4.7%	-	0.6%	1.8%
Kazokoglu et al/ Turkey [15]	2008/48	NA	NA	NA	NA	58.3%	12.5%	12.5%	10.4%	-	-
Smith et al/ USA[16]	2009/527	44.6%	28%	14.4%	12.9%	29%	21%	5%	2%	3%	NA
Paroli et al/ Italy [17]	2009/257	47.8%	19.4%	24.9%	7.8%	12.8%	19.9%	15.1%	2.9%	1.5%	5.8%
Hamade et al/ Saudi Arabia [18]	2009/163	42%	20%	7%	31%	50%	15%	4%	5%	16%	2%

JIA: Juvenile idiopathic arthritis; VKH: Vogt-Koyanagi-Harada disease; NA: not available
